# Psychometric Properties of the Persian Version of Spiritual Well-Being Scale in Patients with Acute Myocardial Infarction

**DOI:** 10.1007/s10943-016-0305-9

**Published:** 2016-09-15

**Authors:** Mohammad Ali Soleimani, Saeed Pahlevan Sharif, Kelly A. Allen, Ameneh Yaghoobzadeh, Hamid Sharif Nia, Ozkan Gorgulu

**Affiliations:** 1 Social Determinants of Health Research Center, Qazvin University of Medical Sciences, Qazvin, Iran; 2 Taylor’s Business School, Taylor’s University, No. 1, Jalan Taylor’s, 47500 Subang Jaya, Selangor Malaysia; 3 The Melbourne Graduate School of Education, The University of Melbourne, Melbourne, Australia; 4 School of Nursing and Midwifery Amol, Mazandaran University of Medical Sciences, Sari, Iran; 5 Department of Biostatistics and Medical Informatics, Faculty of Medicine, Ahi Evran University, Kırşehir, Turkey

**Keywords:** Acute myocardial infarction, Factor analysis, Measurement, Psychometric, Spirituality, Spiritual well-being

## Abstract

The purpose of this study was to assess the psychometric properties of the Persian version of Spiritual Well-Being Scale (SWBS) in patients with acute myocardial infarction. A multisite, cross-sectional survey was employed to determine the instrument’s reliability (Cronbach’s α and construct reliability) and validity (face, content, and construct). Using systematic sampling of adult outpatients at primary care clinic sites in the Qazvin City, Iran (*N* = 300), it was found that the Cronbach’s alpha and construct reliability of both factors associated with the SWBS were above 0.7. The construct validity of the scale was determined using exploratory factor analysis. The findings supported two factors: relation with God and relation with life. Further investigation through confirmatory factor analysis (eigenvalues of greater than one) confirmed a third factor construct associated with the SWBS. A total of 50.65 % of the variance were explained by these three factors. The overall findings of the study demonstrated that the SWBS is a valid and reliable instrument that has potential utility in future research and clinical practice settings.

## Introduction

Psychological stress, anxiety, and loneliness have been found to be associated with non-communicable diseases, including cardiovascular disease (Amiri et al. 2015). Myocardial infarction (MI), commonly referred to as a *heart attack*, is the most common cardiovascular disease (Sharif Nia et al. 2013). Despite extensive diagnostic and treatment developments, one in three patients who suffer AMI result in death following the event (Taghipour et al. 2014). Research has found that between 5 and 10 % of survivors die in the first year following a heart attack. MI has been attributed to physical and psychological problems such as pain, anxiety, and depression (Talebizadeh et al. 2009).

Spiritual well-being (SWB) is described as a complex construct encompassing existential and religious dimensions (Hungelmann et al. 1996). Existential well-being relates to an individual’s endeavor to understand the meaning and purpose of life, while religious well-being refers to the satisfaction gained from a belief in a superior power, such as God (Asarrodi et al. 2011). In other words, SWB refers to the affective experience of positive feelings that result from one’s ability to experience meaning and purpose in life through his or her relationship with oneself, others, and a higher power. SWB has therefore been defined as the “affirmation of life in relationships with oneself (personal), others (communal), nature (environment), and God (or transcendental other)” (National Interfaith Coalition on Aging [NICA] 1975, cited in Gomez and Fisher 2005, p. 1108). Spiritual well-being is achieved through a dynamic processes that involves cognitive, functional, and emotional domains (Abbasi et al. 2013). This aspect of well-being is not limited to prayer or religiosity and may play an instrumental important role in the betterment of disease and use in conventional or complementary medicine. In fact, spiritual vision, values, beliefs, and behaviors have been found to effect the human body at a biochemical and physiological level. Therefore, understanding SWB through empirical measures has important implications for medical interventions and treatment (Marandi and Azizi 2011).

Spirituality can arise in individuals during a time of crisis and stress following the experience of adversity. In these instances, spirituality has been found to reduce tension and stress and increase support and social interaction (Penman et al. 2009). Research has found that SWB increases adaptation abilities and enhances the physical, social, and psychological aspects of good health (Allahbakhshian et al. 2010). SWB has therefore had positive implications for research that has investigated *quality of life* (Asarrodi et al. 2011; Yazdi Moghadam et al. 2009). In fact, this research has demonstrated that high levels of SWB are positively associated with enhanced life satisfaction (Yazdi Moghadam et al. 2009). Thus, given the importance of SWB toward physical and psychological health, there is a need for the psychometric evaluation of measurements evaluating SWB (e.g., the Sense of Well-Being Scale [SWBS]) (Unterrainer et al. 2012).

In the literature, several measures are available to evaluate SWB. The SWBS, one of the most commonly used scales to study SWB, was first developed by Paloutzian and Ellison (1982). The scale consists of two dimensions, namely religious well-being and existential well-being, which measure an individual’s relationship with God as well as life satisfaction, spirituality, and purpose in life, respectively (Ellison 1983; Paloutzian and Ellison 1982). Another popular scale used to measure SWB is the Spiritual Well-Being Questionnaire designed by Gomez and Fisher (2003). This scale assesses four aspects of SWB including personal, social, environmental, and transcendence (Gomez and Fisher 2003). A third measure is the Spiritual Orientation Inventory (Elkins et al. 1988) based on existential and valuable issues. Last, the JAREL Spiritual Well-Being Scale is another tool to measure SWB which includes personal questions about participants, others, and God (Hungelmann et al. 1996). Given that the SWBS is the most widely used measure of SWB in the empirical literature reporting good validity and reliability (Dehshiri et al. 2013), it was deemed the most suitable questionnaire for the current study.

Patients with AMI are exposed to the prospect of death and dying and, as a consequence, may experience depression and anxiety (Sharif Nia et al. 2014). Investigating the role of spirituality in reducing negative psychology sequel has merit for clinical practice and associated interventions (Ebadi et al. 2009). Research has found that spiritual health has a notable role in increasing adaption and reducing stress and anxiety related to death and dying (Jadidi et al. 2011).

Some research has investigated the role that cultural contexts play in the experience of spirituality and SWB. For example, in Western cultures, SWB is a construct considered to be conceptualized through social norms, culture, and religion, often connected to believing in one or multiple gods (Finch 2003; Jha and Singh 2014). Less is understood about how SWB is applied in Iranian cultural. Although some studies have been conducted using the SWBS to evaluate SWB in Iran (Hashemian and Khademi 2015; Mahbobi et al. 2012; Nabatian et al. 2013), no prior research, to the authors’ knowledge, has psychometrically evaluated the SWBS in Iran among patients who have experienced AMI. Therefore, the present study aims to investigate the psychometric properties of SWBS among Iranian patients with AMI and address a significant gap in the empirical literature.

## Methods

### Design

The minimum sample size for conducting factor analysis is equal to 5–10 times more than the number of the items of the intended instrument (Kellar and Kelvin 2012). Consequently, 300 patients via convenience sampling method participated in this study. Participants were referred to the study from Bo Ali Sina and Velayat Hospital in Qazvin, Iran, between August and October 2015. The inclusion criteria for participation in the study included patients having: (1) necessary communication skills, (2) no reported psychological problems such as anxiety and depression in the 4 weeks prior to the study, and (3) stable vital signs. All patients had been hospitalized for at least 24 h prior to participating in the study and were reported to be in a stable condition in respect of vital signs and cardiac hemodynamics. Typically, patients were selected post-discharge from the respective hospitals’ Cardiac Care Units (CCUs).

### Measures

Patients were asked to complete the Persian version of SWBS. The questionnaire consisted of two parts: (1) items eliciting demographic information and (2) the Spiritual Well-Being Scale (SWBS).

#### Demographic Items

The demographic questionnaire contained items relating to age, gender, marital status, level of education, socioeconomic status, primary source of income, experiences of death, social support, and religious beliefs. Perceived level of sociality and religiosity was measured with two items that were developed by nursing researchers. The use of an abbreviated measure was adapted for the study to reduce participant response fatigue. These two items asked the patient to evaluate their amount of religious belief from 1 to 10 (1 = the lowest, 10 = the highest) and their amount of social support from 1 to 10 (1 = the lowest, 10 = the highest) on a Likert-type scale.

#### Spiritual Well-Being Scale

SWBS is a general indicator of perceived well-being which may be used for the assessment of both individual and congregational SWB. It provides an overall measure of an individual’s perception of the spiritual quality within their lives and consists of two subscales: religious well-being and existential well-being. The religious well-being subscale (10 items) provides a self-assessment of one’s relationship with God, while the existential well-being subscale (10 items) offers a self-assessment of one’s sense of life purpose and life satisfaction (Paloutzian and Ellison 1982). SWBS employs a six-point Likert-type scale that ranges from completely disagree (1) to completely agree (6). A reversed scoring method was used for negative questions (items 1, 2, 5, 6, 9, 12, 13, 16, and 18). The range of scores for each of the religious and existential subscales was between 10 and 60. Higher scores indicated higher religious and existential well-being.

The World Health Organization protocol was used to translate and adapt the English SWBS into Persian (World Health Organization 2009). A forward–backward translation technique was used, and accordingly, two English–Persian translators were invited to independently translate the SWBS. An expert panel comprised of the authors of this paper and two translators was used to assess the two translated questionnaires and produce a single Persian version. A Persian–English translator was then asked to back-translate the Persian SWBS into English.

## Validity Assessment

The validity of SWBS was investigated using face validity, content validity, and construct validity.

### Face Validity

The face validity of the Persian SWBS was assessed both qualitatively and quantitatively.

#### Qualitative Face Validity Assessment

To assess the qualitative face validity of the Persian SWBS, ten patients who had experienced AMI were invited to assess and comment on the appropriateness, difficulty, relevance, and ambiguity of the items. The necessary time for completing the scale was determined in this step. The scale was amended according to patient comments.

#### Quantitative Face Validity Assessment

The *item impact technique* was adopted for assessing the quantitative face validity of the Persian SWBS. Ten patients were asked to pilot the scale and determine the importance of the items on a Likert-type scale from 1 (Not important) to 5 (Completely important). The item impact score of each item was calculated by using the following formula, Importance × Frequency (%). In this formula, frequency is equal to the number of patients who had ascribed a score of 4 or 5 to the intended item and importance was equal to scores 4 or 5. If the impact score of the item was greater than 1.5, the item was considered as suitable and it was maintained in the scale (Hajizadeh and Asghari 2011; Maasoumi et al. 2013).

## Content Validity Assessment

The content validity of the Persian SWBS was also assessed both qualitatively and quantitatively.

### Qualitative Content Validity Assessment

In this step, the Persian SWBS was provided to fifteen experts (5 nursing doctorates, 2 psychiatrists, 4 clinical psychologists, and 4 cardiologists) who were asked to assess and comment on the wording, item allocation, and scaling of the items (Colton and Covert 2007). The SWBS was revised according to comments and feedback.

### Quantitative Content Validity Assessment

The quantitative content validity of the scale was assessed through calculating the content validity ratio (CVR) and the content validity index (CVI) for the items. CVR reflects whether the items are essential or not. Accordingly, fifteen experts (see “Qualitative Content Validity Assessment” section) were asked to rate the essentiality of the SWBS items on a three-point scale as follows: Not essential: 1; Useful but not essential: 2; and Essential: 3 (Cook and Beckman 2006). The CVR of each item was calculated by using the following formula: CVR = (*n*_*e*_ − (*N*/2))/(*N*/2). In this formula, *N* and *n*_*e*_ are, respectively, equal to the total number of experts and the number of experts who score the intended item as “Essential.” According to Lawshe (1975), when the number of panelists is 15, the minimum acceptable CVR is equal to 0.49 (Lawshe 1975).

On the other hand, CVI shows the degree to which the items of the intended scale are simple, relevant, and clear. CVI can be calculated for each item of a scale (item level or I-CVI) and for the overall scale (scale level or S-CVI). Accordingly, we asked the same fifteen panelists to rate the simplicity, relevance, and clarity of the SWBS items on a four-point scale from 1 to 4. For instance, the four points for rating the relevance of the items were “Not relevant,” “Somewhat relevant,” “Quite relevant,” and “Highly relevant” which were scored as 1, 2, 3, and 4, respectively. The I-CVI of each item was calculated by dividing the number of panelists who had rated that item as 3 or 4 by the total number of the panelists. Lynn et al. (2006) noted that when the number of panelists is equal to fifteen, the items which acquire an I-CVI value of 0.79 or greater are considered as appropriate (Jay Lynn et al. 2006).

## Construct Validity Assessment

To assess construct validity, the factor structure of the Persian SWBS was examined by conducting an exploratory factor analysis (EFA) by performing a maximum likelihood (ML) followed by a Promax rotation with SPSS 22 (SPSS Inc., Chicago, IL, USA). The Kaiser–Meyer–Olkin (KMO) test and the Bartlett’s test of sphericity were used to check the appropriateness of the study sample and the factor analysis model. The number of factors was determined based on eigenvalues and scree plot. Items with absolute loading values of 0.4 or greater were regarded as appropriate (eigenvalues of one or less should be ignored) (Harrington 2008). The factor structure obtained from the EFA was then examined with using a confirmatory factor analysis (CFA) conducted with AMOS 21. To assess the model fit, several fit indices such as χ2 goodness-of-fit index per degree of freedom (CMIN/*df* < 3), root-mean-square error of approximation (RMSEA < .08), standardized root-mean-square residual (SRMR < .1), goodness-of-fit index (GFI > .9), comparative fit index (CFI > .9), incremental fit index (IFI > .9), and Tucker–Lewis index (TLI > .9) were used (Hooper et al. 2008; Schreiber et al. 2006). Convergent validity and discriminant validity of the factors were evaluated using construct reliability and Fornell–Larcker criterion, respectively. To establish discriminant validity, the square root of the AVE of each factor must be larger than the correlation between two factors (Fornell and Larcker 1981).

## Reliability Assessment

Internal consistency, test–retest analyses, and construct reliability were used to assess the reliability of the Persian version of SWBS. Cronbach’s alpha coefficient was used to assess the internal consistency for which a value α 0.6 is considered acceptable for descriptive studies (Bland and Altman 1997). Cronbach’s alpha coefficient was measured for both individual subscales and the whole questionnaire. Intra-class correlation coefficients (ICCs) were used to establish the test–retest reliability of the SWBS over an interval of 2 weeks using two-way mixed intra-class correlation coefficients (ICCs) for absolute agreement at the level of individual items (Grove and Burns 2005). ICC was also calculated, and its results were interpreted as follows: 0.0–0.2 as low, 0.21–0.40 as fair, 0.41–0.60 as moderate, 0.61–0.80 as substantial, and 0.81–1 as almost perfect (Landis and Koch 1977). Construct reliability of the extracted factors was assessed by following Hair et al.’s (2010) approach. Construct reliability greater than .7 demonstrates good reliability (Hair et al. 2013).

## Ethical Considerations

The study was approved by the Qazvin University of Medical Sciences Ethics Committee (QUMS.REC.1394.10). Patients were informed about study aims and procedures that participation was voluntary, and would not affect medical care before signing an informed consent document. Patient confidentiality was assured by completing all study procedures in a quiet treatment area. To ensure that a broad cross section of patients was allowed to participate in the study, a trained research assistant provided support as needed. All personal data were de-identified with the use of assigned codes.

## Results

An overall survey response rate of 81 % was reported for the present study. Table 1 describes the demographic profiles of the respondents. The respondents were predominately married (*n* = 244, 81.3 %) with a mean age of 59.89 years. Among them, 57.3 % (*n* = 157) were female.

**Table 1:** Demographic characteristics of the study participants

Demographic characteristics	Number (%)
Sex	
Male	143 (47.7)
Female	157 (57.3)
Marriage	
Single	5 (1.7)
Married	244 (81.3)
Widowed	51 (17)
Employment	
Yes	119 (36.1)
No	211 (63.9)
Educational status	
No formal education	155 (51.7)
Primary	69 (33)
Intermediate	30 (10)
High school	36 (12)
Collegiate	10 (3.3)
Economic status	
Poor	71 (23.7)
Average	213 (71.0)
Good	16 (5.3)
Main source of income	
Personal	129 (43)
Family	26 (8.7)
Friends	3 (1)
Pension from the government	117 (39)
Charity	25 (8.3)
Death experiences	
Yes	18 (6)
No	282 (94)
	Mean (SD), range
Age	
Age of subject	59.89 (11.94), 22–96
Social support	6.32 (2.76), 0–10
Religious belief	9.08 (1.47), 0–10
Spiritual well-being	
Total SWB	96.24 (11.84), 40–116
Religious well-being	57.81 (5.70), 10–60
Existential well-being	38.38 (9.24), 16–56

The impact score, CVR, and I-CVI values of all the 20 items of the Persian SWBS were, respectively, greater than 1.5, 0.49, and 0.79 (Table 2), and hence, none of the items were excluded in these steps of psychometric evaluation.

**Table 2:** CVR and I-CVI for the TDAS items

No.	Items	CVI			CVR
		Simplicity (1–4)	Relevancy (1–4)	Clarity (1–4)	Essential (1–3)
1	I don’t find much satisfaction in private prayer with God	.86	.8	.93	.86
2	I don’t know who I am, where I came from, or where I’m going	.86	1	.8	1
3	I believe that God loves me and cares about me.	.86	.93	.8	.86
4	I feel that life is a positive experience	.86	1	.93	1
5	I believe that God is impersonal and not interested in my daily situations	1	1	1	1
6	I feel unsettled about my future	.86	.93	.8	1
7	I have a personally meaningful relationship with God	.86	.8	.8	.86
8	I feel very fulfilled and satisfied with life	.93	1	.93	1
9	I don’t get much personal strength and support from my God	.93	1	.8	.86
10	I feel a sense of well-being about the direction my life is headed in	.86	1	.93	1
11	I believe that God is concerned about my problems.	.86	1	.8	.86
12	I don’t enjoy much about life	1	1	1	1
13	I don’t have a personally satisfying relationship with God	.86	1	.86	1
14	I feel good about my future	.86	.93	.86	1
15	My relationship with God helps me not to feel lonely	.86	1	.8	1
16	I feel that life is full of conflict and unhappiness	.93	1	1	1
17	I feel most fulfilled when I’m in close communion with God	1	1	1	1
18	Life doesn’t have much meaning	1	.93	.93	1
19	My relation with God contributes to my sense of well-being	1	1	1	1
20	I believe there is some real purpose for my life	1	1	.93	.86

**Fig. 1:**
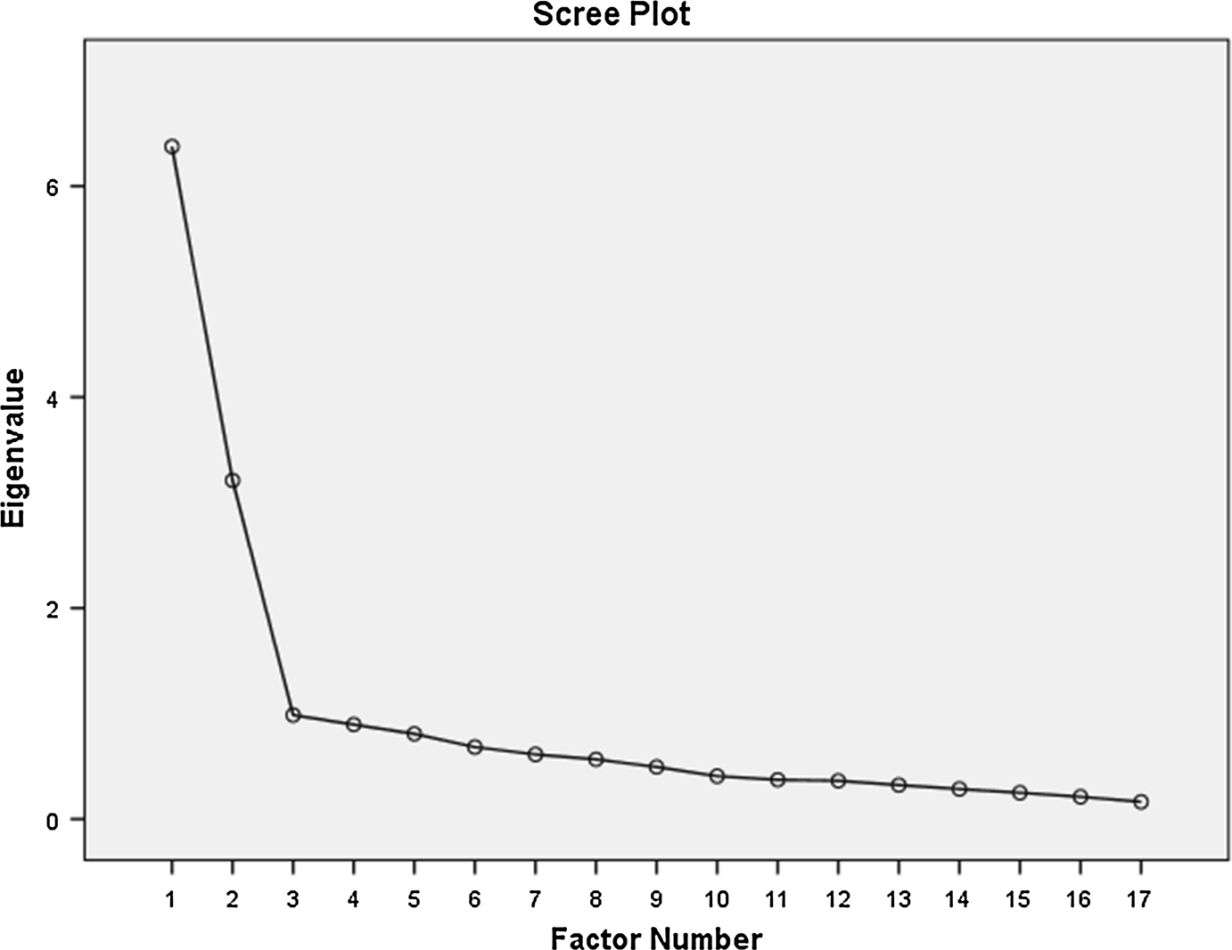
Scree plot of SWBS among AMI patients

The value of Kaiser–Meyer–Olkin (KMO) was .879, indicating that the data and sample size were adequate for factor analysis. Moreover, the approximate Chi-square value of Bartlett’s test of sphericity (*χ* ^*2*^ = 2785.408, *df* = 136, *p* < .001) confirmed that the factor model is appropriate. These two tests showed the suitability of the respondent data for exploratory factor analysis. Exploratory factor analysis using maximum likelihood method with Promax rotation was performed on the 20 items of the Persian version of the SWB scale. As shown in Table 3, based on scree plot (Fig. 1), the two factors that reported eigenvalue greater than 1, accounting for 50.650 % of the variance, were extracted. Three items (2, 6, and 20) were deleted from the model as they had factor loadings less than .5.

**Table 3:** Exploratory factor loadings of items in the SWB with two factors

Item No.	Factors of attitude sub-questionnaire	Mean	SD	*h* ^2^	Factor loading	
					Factor 1	Factor 2
Factor 1: Relation with God (% of variance = 34.738, eigenvalue = 5.906)						
9	I don’t get much personal strength and support from my God	5.78	0.81	.696	**.808**	.084
11	I believe that God is concerned about my problems	5.78	0.73	.647	**.793**	.039
19	My relation with God contributes to my sense of well-being	5.84	0.56	.611	**.792**	−.045
7	I have a personally meaningful relationship with God	5.69	0.82	.624	**.789**	.002
13	I don’t have a personally satisfying relationship with God	5.82	0.71	.603	**.776**	.002
17	I feel most fulfilled when I’m in close communion with God	5.74	0.69	.603	**.769**	.024
3	I believe that God loves me and cares about me.	5.80	0.70	.550	**.706**	.106
15	My relationship with God helps me not to feel lonely	5.76	0.83	.409	**.658**	−.090
1	I don’t find much satisfaction in private prayer with God	5.80	0.81	.341	**.605**	−.135
5	I believe that God is impersonal and not interested in my daily situations	5.80	0.80	.359	**.592**	.025
Factor 2: Relation with Life (% of variance = 15.910, eigenvalue = 2.705)						
4	I feel that life is a positive experience	4.10	1.36	.627	−.202	**.822**
16	I feel that life is full of conflict and unhappiness.	3.65	1.37	.541	−.098	**.756**
12	I don’t enjoy much about life	4.42	1.63	.470	.048	**.671**
14	I feel good about my future	4.45	1.47	.426	−.029	**.660**
10	I feel a sense of well-being about the direction my life is headed in	4.89	1.38	.363	.036	**.591**
8	I feel very fulfilled and satisfied with life	4.20	1.47	.383	.173	**.550**
18	Life doesn’t have much meaning	4.51	1.81	.357	.152	**.539**

*SD* standard deviation, *h* ^*2*^ communality

Factor loadings greater than .5 are in bold type

**Fig. 2:**
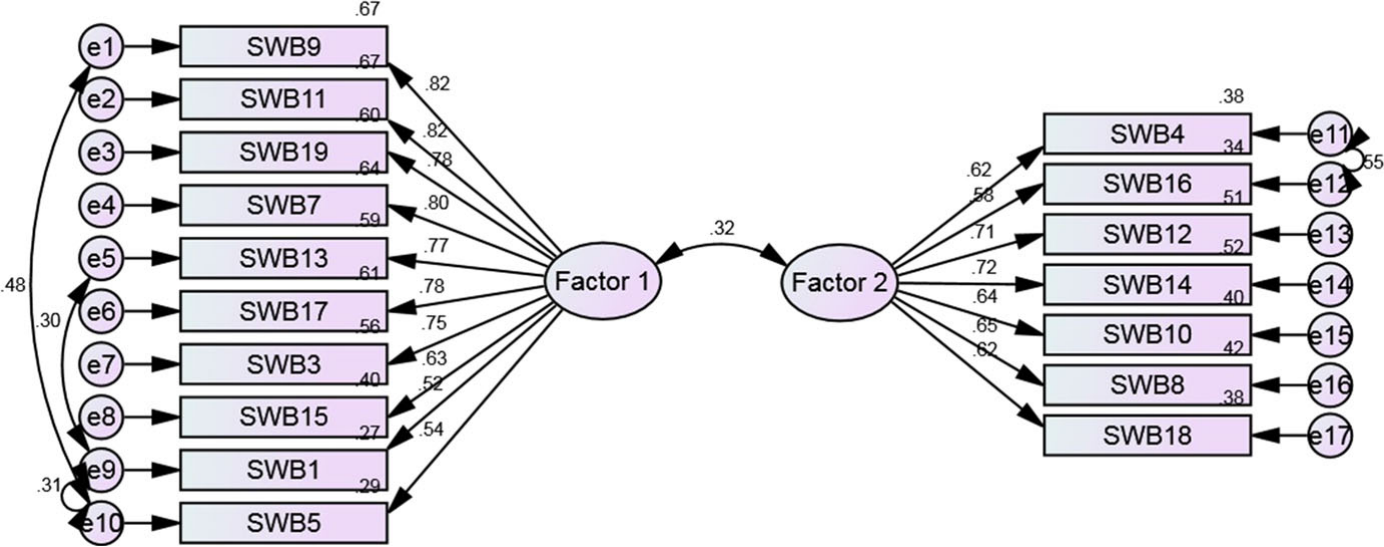
Measurement model of SWB among AMI patients. Note: χ^2^(114) = 330.222, *p* < .05, χ^2^/*df* = 2.897, GFI = .885, CFI = .920, IFI = .921, TLI = .905, standardized RMR = .066, RMSEA (90 % C.I.) = .08 (.070–.090)

Next, CFA was used to confirm and validate the factor structure obtained from EFA. The final model (Fig. 2) was reported after reviewing model modification indices for sources of model misfit. Three pairs of items measurement errors of Factor 1 and one pair of Factor 2 were allowed to freely covary to improve the measurement model fit.

The goodness-of-fit indexes suggested that the revised model had a good fit to the data χ^2^(114) = 330.222, *p* < .05, χ^2^/*df* = 2.897, GFI = .885, CFI = .920, IFI = .921, TLI = .905, standardized RMR = .066, RMSEA (90 % C.I.) = .08 (.070–.090). Moreover, all factor loadings were greater than .5 and significant (*z* value range 8.481–16.423). Following the modification indices, four correlations were inserted between the errors (e1–e10, e5–e9, e9–e10, e11–e12). The results of construct validity assessment are shown in Table 4.

**Table 4:** Construct reliability and Fornell–Larcker criterion

	Cronbach alpha	Construct reliability	Factor 1	Factor 2
Factor 1	.915	.917	.727	
Factor 2	.840	.835	.321	.649

The diagonal elements of the Fornell–Larcker criterion part are squared root of average variance extracted of the factors, and the non-diagonal elements represent the correlations between the factors

The average measure ICC was .825 with a 95 % confidence interval from .795 to .853 (F (299, 19) = 337.78, *p* < .001). Cronbach’s alpha and construct reliability of both Factor 1 (α = .915, CR = .917) and Factor 2 (α = .840, CR = .835) imply good reliability and convergent validity. In addition, correlation between the two factors is less than the squared root of average variance extracted of the factors, which fulfills the requirements of discriminant validity.

## Discussion

This present study was conducted with the aim of translating and evaluating the reliability and validity of the Persian version of 20-item SWBS among patients who had experienced AMI. Exploratory factor analysis showed that the SWBS is a two-domain structure among AMI patients. The findings of the present study demonstrated that the SWBS is an instrument with two factors, *relation with God, and relation with life*, respectively. The two-component structure has been supported by previous research (Darvyri et al. 2014). Other studies confirmed two factors (e.g., “experience of a relationship with God” and “existential elements of spirituality, in particular, life satisfaction, life direction and future, and life purpose” (Musa and Pevalin 2012) three factors (e.g., “Affiliation with God,” “Satisfaction with life,” and “Alienation from God”—sense of meaningless life [(Darvyri et al. 2014), and four factors (e.g., “Develop trust in others,” “Develop self-awareness,” and “Develop a life of meditation and/or prayer” (Gouveia et al. 2012b) of the present scale. The present results may be explained through the Iranian cultural context where the study took place. The presence of a predominantly Muslim community means that spirituality is a central aspect of life to most participants in the study. The cultural and religious context of a society may influence individuals, both corporeally and spiritually (Sharif Nia et al. 2015).

One of the two factors identified in the EFA was the relation with God. This type of relationship together with spirituality has been positively correlated with a higher life expectancy (Sharif Nia et al. 2012). A strong relationship with God can decrease anxiety, stress, and frustration (Lyon et al. 2014). Although an AMI event may occur suddenly, the type of relationship one has with God may affect how individuals cope and recover after life threatening events (Blumenthal et al. 2007). Various studies have assessed different types of relationships with God and SWB, as well as dependent variables like anxiety, death anxiety, hope, and sense of purpose in life in patients with chronic illness (Herth 2000; Kim and Yong 2013; Lyon et al. 2014; Rezaie Shahsavarloo et al. 2015). However, to the author’s knowledge, no research has been conducted that has specifically evaluated SWB with AMI to compare the results of the present study.

The results of an EFA also showed reported a second factor associated with quality of life. Acute and chronic conditions are often accompanied by a great deal of psychological distress and mood disturbance affecting the life and well-being of individuals (Sawatzky et al. 2007). Thus, following a heart attack, achievement of life goals across past, present, and future seems to contribute toward the extent of life satisfaction reported by participants (Baldacchino 2014). Alongside the negative consequences of AMI, positive change may also occur. For example, a patient who has experienced AMI may show greater appreciation for life, adopt healthier attitudes toward diet and exercise, value close relationships more, and demonstrate adaptability to their illness (Afrasiabifar et al. 2011). Research shows that patients who have experienced AMI may also deal with the emotional consequences of the illness through mourning the loss of health, experiencing anxiety, and expressing uncertainty about the future (Ernstsen et al. 2016). Therefore, in order to understand the relative contributions of several factors affecting patients’ well-being and life satisfaction, all types of well-being, especially spirituality, should be considered in treatment and care options (Baldacchino 2014; Shukla and Rishi 2014). Some research has confirmed the positive effect of SWB in increasing quality of life, yet negatively it is correlated with helplessness/hopelessness, and anxious preoccupation (Cotton et al. 1999; Rawdin et al. 2013). Spirituality is considered as an endurance factor in dealing with life threatening conditions (Sharif Nia et al. 2015). On the other hand, individuals with higher SWB are not as concerned about the end of life compared with individuals with low SWB. In fact, these participants report less hopelessness (Rezaie Shahsavarloo et al. 2015). According to the context of Iranian society, for those individuals who are Muslim, a belief in the afterlife lessens anxiety and concern about death (Khezri et al. 2015).

CFA model was used in order to determine the validity of the Persian version of the SWBS. It confirmed the final factor construct of SWBS. In past studies, only two-factor models have been identified: Kirschling and Pittman (1989) used data from 70 caregivers for terminally ill hospice patients (Kirschling and Pittman 1989), Genia (2001) used a religious university student sample (Genia 2001), and Fernander et al. (2004) used data from 661 male prisoners with prior histories of drug use (Fernander et al. 2004). Although studies had confirmed the structural fit of the aforementioned model, the model did not have a suitable factor structure in the African-American population (Shawn et al. 2005).

Cronbach’s alpha and CR of the SWBS showed that the Persian version of the SWBS has good reliability among patients who have experienced AMI, especially when the reliability was calculated separately for each factor. The reliability of the SWBS in various studies using different methods was in line with the present study. For example, Gouveia et al. (2012a) reported that the internal consistency of the four discovered factors was higher than 0.7. Moreover, Paloutzian and Ellison assessed the internal consistency of the SWBS using Cronbach’s alpha coefficient and the reliability was calculated as 0.88 (Paloutzian and Ellison 1982). Research conducted in Iran among patients with chronic conditions showed that the internal stability was considered favorable (Seyed fatemi et al. 2006). In fact, the Cronbach’s alpha value indicates good internal consistency of the scale and it also shows an adequate correlation between the items employed. Therefore, it can be assumed that the items that comprise the scale assess similar concepts.

The present study has several limitations. Spirituality is a nebulous construct, and the purpose of the study was to evaluate the psychometric properties of an instrument gauging something arguably ill-defined. As a result, the present study was unable to analyze or report normative data about the SWBS. According to the final model of SWBS of the current study, participants’ item responses are affected by measurement errors. Moreover, it was found that the error terms are correlated, as indicated by the curved lines connecting the error. Munro (2005) states that correlated measurement error occurs in situations where variables have not been clearly identified, defined, or measured directly, which can affect how participants respond to certain items (Munro 2005). A latent variable (a variable that is not directly observed) consists of only the true scores of a construct’s indicators and is therefore not subject to measurement error (Jiang 2014). Measurement errors may be caused by a study’s methodology (e.g., self-report questionnaires). On the other hand, measurement errors can result from words that have a similar meaning and phrases in both positive and negative statements (Harrington 2008). Correlated measurement error might arise as a result of respondent’s desire to agree with factors that would orderly affect all item scores (Polit and Beck 2008). The present study is vulnerable to each of these types of errors.

## Conclusions

In summary, the SWBS is a valid and reliable measure for assessing SWB among patients who have experienced AMI. Therefore, the Persian version of SWBS can be used as a valid and reliable tool for the assessment of SWB in patients who have faced an AMI event. Future validation studies with multiple populations and longitudinal designs are needed to refine, modify, or verify the SWBS as an additional, complementary instrument of well-being. Moreover, the development of a more appropriate measure of spirituality may be warranted in order to enhance future study in the area of spirituality and health research.

## Appendix

Persian version of Spiritual Well-Being Scale.
